# The Role of PRRC2B in Cerebral Vascular Remodeling Under Acute Hypoxia in Mice

**DOI:** 10.1002/advs.202300892

**Published:** 2023-07-03

**Authors:** Shuoshuo Li, Wenyu Hu, Shenghui Gong, Ping Zhang, Jinbo Cheng, Shukun Wang, Yingyi Wang, Wenjun Shi, Qianqian Li, Fengchao Wang, Zengqiang Yuan

**Affiliations:** ^1^ School of Life Science Beijing University of Chinese Medicine Beijing 100029 China; ^2^ The Brain Science Center Beijing Institute of Basic Medical Sciences Beijing 100850 China; ^3^ School of Medicine University of South China Hengyang 421001 China; ^4^ Center on Translational Neuroscience College of Life & Environmental Science Minzu University of China Beijing 100081 China; ^5^ National Institute of Biological Sciences Beijing 102206 China

**Keywords:** cognitive defect, COL12A1, hypoxia, N6‐methyladenosine, proline‐rich coiled‐coil 2B, vascular remodeling

## Abstract

High altitude exposure leads to various cognitive impairments. The cerebral vasculature system plays an integral role in hypoxia‐induced cognitive defects by reducing oxygen and nutrition supply to the brain. RNA N6‐methyladenosine (m6A) is susceptible to modification and regulates gene expression in response to environmental changes, including hypoxia. However, the biological significance of m6A in endothelial cell performance under hypoxic conditions is unknown. Using m6A‐seq, RNA immunoprcipitation‐seq, and transcriptomic co‐analysis, the molecular mechanism of vascular system remodeling under acute hypoxia is investigated. A novel m6A reader protein, proline‐rich coiled‐coil 2B (PRRC2B), exists in endothelial cells. PRRC2B knockdown promoted hypoxia‐induced endothelial cell migration by regulating alternative splicing of the alpha 1 chain of collagen type XII in an m6A‐dependent manner and the decay of matrix metallopeptidase domain 14 and ADAM metallopeptidase domain 19 mRNA in an m6A‐independent manner. In addition, conditional knockout of PRRC2B in endothelial cells promotes hypoxia‐induced vascular remodeling and cerebral blood flow redistribution, thus alleviating hypoxia‐induced cognitive decline. Therefore, PRRC2B is integral in the hypoxia‐induced vascular remodeling process as a novel RNA‐binding protein. These findings provide a new potential therapeutic target for hypoxia‐induced cognitive decline.

## Graphical abstract

This study identifieds a novel m6A reader protein, proline‐rich coiled‐coil 2B (PRRC2B), in cerebral endothelial cells. PRRC2B knockdown alters the pre‐mRNA splicing of COL12A1 in human umbilical vein endothelial cells (HUVECs) and promotes endothelial cell migration. Conditional knockout of PRRC2B in cerebral endothelial cells alleviates hypoxia‐induced cognitive decline by supplying a remodeling system and regulating cerebral blood flow.

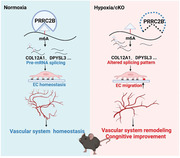

## 1 Introduction

Acute exposure to high altitude causes cognitive impairment. High altitude severely affects cognitive functions due to decreased oxygen intake.^[^ ^1^ ^]^ The brain is one of the highest oxygen‐consuming organs in the mammalian body because of its higher oxidative phosphorylation level. Thus, the brain is sensitive to hypoxia.^[^ ^2^, ^3^ ^]^ The cerebral vasculature system supplies oxygen and nutrients to the brain. Under hypoxic conditions, the vascular system responds to oxygen shortage and redistributes blood flow, thus ensuring oxygen supply for critical tissues/organs.^[^ ^4^, ^5^ ^]^ Reduced available oxygen activates vascular endothelial growth factor production, which stimulates angiogenesis to restore oxygen and nutrient supply to hypoxic areas.^[^ ^6^ ^]^ Recent studies have revealed that during hypoxia stimulation, the capillaries or vessel terminals are regenerated, and the vascular system is also subjected to remodeling.^[^ ^7^ ^]^ However, the underlying mechanism of hypoxia‐induced cerebral vascular remodeling remains unclear.

Epigenetic regulation, especially post‐transcriptional RNA regulation, is essential in hypoxia‐induced brain injury.^[^ ^8^, ^9^ ^]^ N6‐methyladenosine (m6A) is a methylation modification of the RNA base adenosine. It widely exists in eukaryotic cells and is vital in a hypoxic environment.^[^ ^10^ ^]^ Recent evidence indicates that m6A is abundant in the brain and participates in several biological processes. Multiple readers and functions of m6A have been identified in previous studies in neuron and glial cells,^[^ ^11^, ^12^ ^]^ but have rarely been studied in cerebral endothelial cells.

Vascular structure modeling is mainly determined by the growth, death, and migration of endothelial cells and the production or degradation of the extracellular matrix (ECM).^[^ ^13^ ^]^ Evidence reveals that aberrant m6A modifications are involved in vasculogenesis and angiogenesis in embryo development, which indicates the functional importance of m6A in endothelial cells.^[^ ^14^ ^]^ However, the mechanism through which m6A regulates endothelial cell performance and participates in vascular remodeling in a hypoxic environment has not been determined. Therefore, this study aimed to illustrate the proline‐rich coiled‐coil 2B (PRRC2B)‐mediated mechanisms of hypoxia‐induced cognitive decline.

Here, we identified a novel m6A‐specific binding protein, PRRC2B, in cerebral endothelial cells. We revealed the target genes of PRRC2B by RNA immunoprecipitation (RIP)‐seq, m6A seq, and transcriptomic conjoint analysis, and demonstrated the role of PRRC2B in endothelial performance by knockdown strategies in human umbilical vein endothelial cells (HUVECs). In addition, we illustrated the effect of PRRC2B on hypoxia‐induced cognitive defects in vivo by conditional PRRC2B knockout in cerebral endothelial cells.

## 2 Results

### 2.1 PRRC2B is a Novel m6A Reader that is Expressed and Functional in Cerebral Endothelial Cells

**Figure 1:**
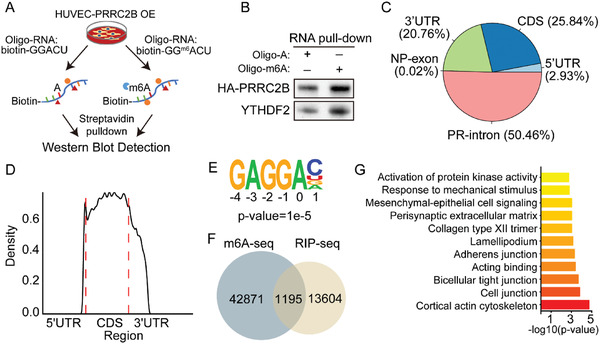
PRRC2B is a novel m6A reader expressed and functional in cerebral endothelial cells. A) Schematic illustration of m6A probe pull‐down and detection of m6A binding protein in a PRRC2B‐overexpressing HUVEC single clone. B) Western blot showing PRRC2B and YTHDF2 pulled down with an m6A‐containing RNA probe. YTHDF2 is an m6A binding protein, here as a positive control. C) Pie chart depicting the distribution of PRRC2B binding peaks in PRRC2B RIP‐seq. D) Distribution of PRRC2B peaks across the length of the mRNA. E) PRRC2B binding motif identified by HOMER, *p* = 1e‐5. F) Overlap of m6A modification peaks and PRRC2B binding peaks. G) Gene Ontology (GO) is a biological process enriched in the intersection of genes in (F).

PRRC2B is a member of the PRRC2 protein family. Previous research demonstrated that PRRC2A and PRRC2C are m6A readers in oligodendrocytes.^[^ ^11^ ^]^ To determine whether PRRC2B also functions as an m6A reader in cerebral endothelial cells owing to the structural similarity between PRRC2A and PRRC2B, we created a stable HUVEC single clone line overexpressing 3 × FLAG‐PRRC2B‐HA using lentivirus. Using a methylated RNA bait with known consensus sites of G(m6A)C versus an unmethylated control in cell lysates, we identified PRRC2B as a potential m6A binding protein (**Figure** **1**A,B). We performed PRRC2B RIP‐seq and m6A RIP‐seq in HUVECs to further demonstrate that PRRC2B binds to m6A‐containing RNA (Figure S1A,B, Supporting Information). In the PRRC2B RIP‐seq data, we identified 14 799 PRRC2B binding peaks within 4375 genes, with most of the peaks located in mRNAs (49.53%) and pre‐RNAs (50.46%) (Figure 1C). The enrichment of PRRC2B binding peaks in 3ʹUTR and CDS is consistent with the previously reported pattern of m6A peaks (Figure 1D and Figure S1C,D, Supporting Information). Additionally, our analysis of the PRRC2B binding motif revealed that 30.46% of the binding sites are GAGGAC (Figure 1E 1), which overlaps with the canonical m6A motif (Figure S1E, Supporting Information), indicating the enrichment of m6A at PRRC2B binding positions. The distribution features of m6A peaks identified in HUVECs were consistent with previous reports,^[^ ^11^ ^]^ supporting the reliability of our m6A‐seq data (Figure S1C–E, Supporting Information). Most genes associated with PRRC2B binding have m6A modification (Figure S1F, Supporting Information). We overlaid the PRRC2B binding peaks with m6A peaks and observed that 8% (1195/14 799) of the PRRC2B peaks overlapped with m6A (Figure 1F), indicating that PRRC2B binds directly to m6A regions. To further demonstrate the function of PRRC2B, we performed GO analysis of the genes associated with PRRC2B and m6A overlapped peaks, revealing enrichment in vessel system organization and structure modeling processes (Figure 1G). These results suggest that PRRC2B is a novel m6A reader in endothelial cells and that its binding to m6A modification is important in regulating endothelial cell function.

**Figure 2:**
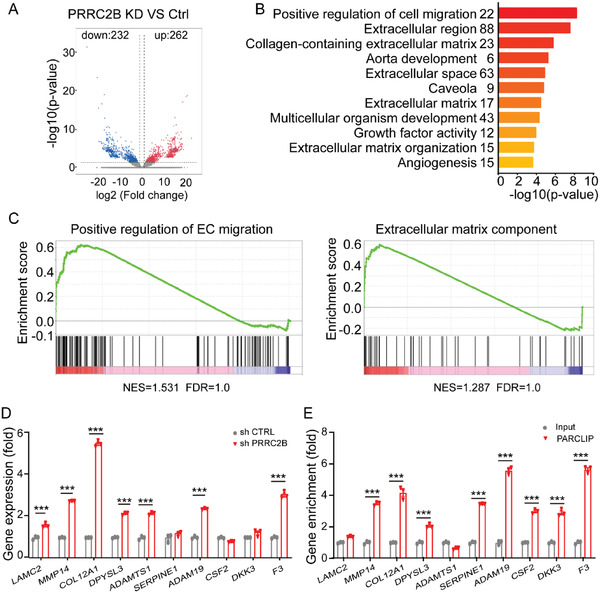
PRRC2B knockdown promotes the expression of genes mainly enriched in the positive regulation of endothelial cell migration and ECM components. A) Volcano plot of transcriptomic data reveals the different gene expressions in PRRC2B‐knockdown HUVECs versus control. B) Gene Ontology (GO) terms of the biological process categories enriched in differential expression genes in RNA‐seq data. C) GSEA plot reveals that the genes enriched in endothelial cell migration and extracellular components were upregulated in PRRC2B‐knockdown HUVECs. D) Relative gene expression in PRRC2B‐knockdown HUVECs. E) PRRC2B directly binds to cell migration regulation and extracellular matrix‐related gene mRNA by PRRC2B PAR‐CLIP‐qPCR analysis. (*N* = 3 in QPCR experiment, Student's *t*‐test, *p < 0.05, **p < 0.01, ***p < 0.001).

To further elucidate the downstream genes of PRRC2B in endothelial cells, we performed RNA‐seq analysis in HUVECs. Our findings revealed that PRRC2B knockdown in HUVECs upregulated 262 gene expressions and downregulated 232 gene expressions (**Figure** **2**A). GO analysis revealed that the differentially expressed genes (DEGs) were mainly enriched in cell migration regulation and extracellular regions (Figure 2B). Consistently, Gene Set Enrichment Analysis (GSEA) revealed that the upregulated genes in PRRC2B knockdown were mainly enriched in the positive regulation of endothelial cell migration and ECM components (Figure 2C). To validate these results, we performed a qPCR assay to examine the expression levels of the genes involved in cell migration regulation and ECM components. Our results were consistent with the RNA‐seq data (Figure 2D). Furthermore, we performed a PAR‐CLIP assay followed by qPCR to validate whether PRRC2B directly binds to those gene mRNAs, and the results show that PRRC2B directly binds to ECM‐related genes (Figure 2E). These results indicate that PRRC2B knockdown promotes the expression of genes mainly enriched in the positive regulation of endothelial cell migration and ECM components.

**Figure 3:**
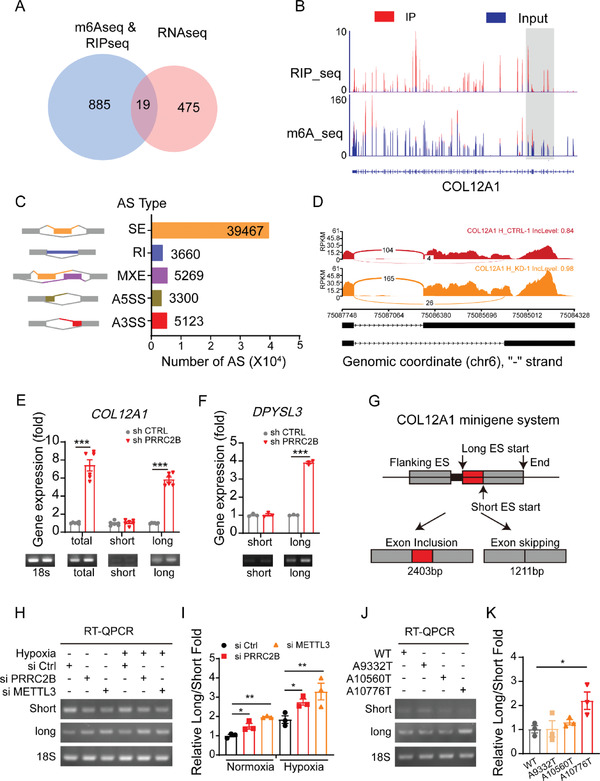
PRRC2B knockdown altered COL12A1 mRNA splicing in m6A dependent manner. A) Venn plot illustrates the overlap of PRRC2B binding peaks containing m6A modification and different expression genes of PRRC2B‐knockdown HUVECs versus control. B) Integrative Genomics Viewer (IGV) tracks displaying PRRC2B RIP‐seq (Up panel), and m6A‐seq (bottom panel) read distributions in COL12A1 mRNA (3ʹ part). Significant peaks are indicated with highlights. C) Differentially expressed alternative splicing events in PRRC2B‐knockdown HUVECs versus the control group. D) Exemplary sashimi plots showing concerted alternative splicing changes that occurred in COL12A1 mRNA in PRRC2B knockdown HUVEC. E) COL12A1 and F) DPYSL3 transcription variant expression levels detected by q‐PCR and agarose gel electrophoresis. G) Schematic of the construction of the COL12A1 mRNA A3SS minigene system. H) RT‐PCR results and I) statistical analysis of the inclusion level of cassette exons upon PRRC2B, or METTL3 knockdown or hypoxia relative to control. J) RT‐PCR results and K) statistical analysis of the inclusion level of cassette exons upon potential m6A site mutations. The inclusion level was quantified and shown as mean ± SEM. (*N* = 3 in QPCR experiment, Student's *t*‐test, *p < 0.05, **p < 0.01, ***p < 0.001).

We then aligned the PRRC2B binding genes containing m6A peaks with the DEGs of PRRC2B‐knockdown HUVECs to determine whether PRRC2B directly regulates those gene fates in an m6A‐dependent fashion. Our results revealed that 19 genes were potentially regulated by PRRC2B in an m6A‐dependent manner (**Figure** **3**A). Importantly, Integrative Genomics Viewer (IGV) tracks display the colocalization of m6A and PRRC2B binding peaks within the alpha 1 chain of collagen type XII (COL12A1) mRNA transcript (Figure 3B). Therefore, we argue that COL12A1 might act as an important target of PRRC2B. m6A‐dependent RNA fate decision regulation has been reported at various levels, including RNA splicing, RNA decay, RNA translocation, and translation.^[^ ^15^ ^]^ To demonstrate the effect of PRRC2B on target gene fate decisions, we performed co‐immunoprecipitation and identified potential PRRC2B binding proteins by mass spectrometry (Figure S2A–C, Supporting Information). GO and KEGG analysis revealed that PRRC2B binding proteins were mainly enriched in the RNA processing pathway, particularly the RNA splicing process (Figure S2D,E, Supporting Information). We then analyzed the transcriptome data and observed that PRRC2B knockdown increased alternative splicing events (Figure 3C). It has been reported that the expression of COL12A1 could be regulated via alternative splicing,^[^ ^16^ ^]^ and in our RNA‐seq data, we found PRRC2B knockdown increased COL12A1 alternative 3ʹ splice site (A3SS) events (Figure 3D). Therefore, we propose PRRC2B may regulate COL12A1 alternative splicing in an m6A‐dependent manner. To validate this hypothesis, we examined the levels of target gene transcription variants by qPCR. We discovered that PRRC2B knockdown increased COL12A1 and DPYSL3 long transcription variant levels (Figure 3E,F). These results indicate that PRRC2B may regulate alternative splicing of target genes in endothelial cells. We investigated the role of m6A modification in regulating COL12A1 expression in METTL3‐knockdown HUVECs, and the results revealed that inhibiting m6A modification increased the expression of long transcriptional variants but did not affect the short variant (Figure S2F,G, Supporting Information). Furthermore, in order to decipher the mechanism of PRRC2B regulation COL12A1 alternative splicing, we construct a minigene system of COL12A1 by cloning the 3ʹ sequence into the pSpliceExpress vector (Figure 3G).^[^ ^17^ ^]^ The splicing pattern of the COL12A1 minigene was examined by knocking down PRRC2B, or METTL3 in HeLa cells (Figure 3H,I). Interestingly, the results in vitro are consistent with the endogenous splicing pattern of COL12A1 in PRRC2B knockdown HUVECs (Figure 3C). Furthermore, to determine whether PRRC2B directly regulates COL12A1 splicing, we generated a series of minigene mutations in the potential m6A modification sites of the COL12A1 minigene and found that the A10776T mutation increased the level of long transcriptional variants (Figure 3J,K) These data clearly demonstrated a direct role of m6A reader PRRC2B in COL12A1 mRNA‐splicing regulation. These results indicated that the m6A modification at 10776A mainly mediated the alternative splicing of COL12A1. Taken together, these data clearly demonstrated a direct role of the m6A reader PRRC2B in the regulation of COL12A1 mRNA‐splicing. Using PRRC2B‐RIP seq/m6A seq/transcriptomic data, we observed that PRRC2B knockdown increased the expression levels of ADAM19 and MMP14; further, we found that PRRC2B also directly binds to its mRNA. However, we failed to find these genes in the intersections of PRRC2B binding and m6A peaks. Further analysis revealed that PRRC2B knockdown increased the mature mRNA levels but had no effect on pre‐mRNA levels of ADAM19 and MMP14. The mature mRNA level was also unchanged in METTL3‐knockdown HUVECs (Figure S2H–J, Supporting Information). These findings suggest that PRRC2B knockdown may increase the mRNA half‐lives of these genes. We validated this hypothesis by performing an mRNA decay assay in PRRC2B‐knockdown HUVECs and discovered that it significantly slowed down mRNA decay (Figure S2K,L, Supporting Information). Thus, PRRC2B knockdown stabilizes MMP14 and ADAM19 mRNA in an m6A‐independent manner. Our results revealed that PRRC2B knockdown increased ECM‐related gene expression by regulating mRNA alternative splicing and decay processes.

We also investigated whether PRRC2B is involved in hypoxia‐induced vascular changes and whether it is highly correlated with endothelial ECM composition. We exposed HUVECs to hypoxic conditions and observed that hypoxia exposure downregulated endogenous PRRC2B expression at both the mRNA and protein levels (Figure S3A,B, Supporting Information). We further validated this phenomenon in vivo by exposing 8‐week‐old mice to a hypobaric and hypoxic environment, followed by dissection of brain parenchymal cells and separation of cerebral endothelial cells as CD45‐CD31+ cells (Figure S3C–E, Supporting Information). Our results revealed that hypoxia treatment decreased PRRC2B expression levels in cerebral endothelial cells in vivo (Figure S3F,G, Supporting Information). Moreover, knockdown or conditional knockout of PRRC2B in endothelial cells increased the expression levels of Col12a1 and Dpysl3 (Figure S3H,I, Supporting Information), indicating that PRRC2B regulates ECM‐related gene expression changes that may function in endothelial cells in response to hypoxic stimulation.

### 2.2 PRRC2B Knockdown Promotes HUVEC Migration

**Figure 4:**
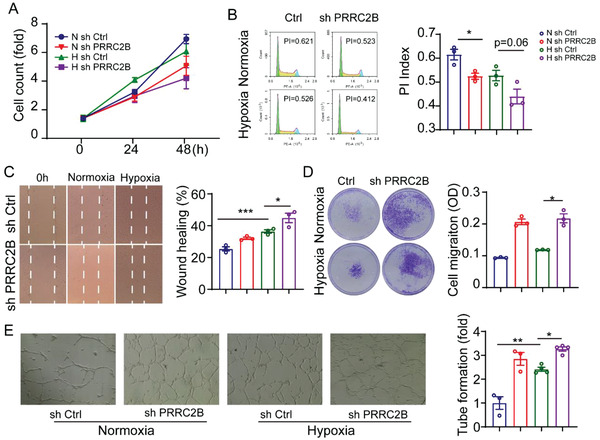
PRRC2B knockdown promotes cell migration. A) The proliferation curve was drawn by cell number counting and the data was shown as a fold change normalized to the control group. (N sh Ctrl vs N sh PRRC2B, *p* = 0.048, H sh Ctrl vs H sh PRRC2B, *p* = 0.11, two‐way ANOVA, *N* = 5). B) Cell cycle detection by flow cytometry with PI staining, statistic data of proliferation index (PI index = (S+G2/M)/(G1+S+G2/M)). C) Wound healing cell migration study of PRRC2B‐knockdown HUVECs in normoxic and hypoxic conditions. D) Transwell migration study of HUVECs in hypoxic conditions. E) Tube formation assay of HUVECs in vitro, control, and PRRC2B knockdown in normoxia and hypoxia, respectively (data in [B–E] were shown as mean ± SEM and normalized to the control group, *N* = 3, Student's *t*‐test, *p < 0.05, **p < 0.01, ***p < 0.001).

The endothelial ECM modulates several endothelial cell processes, including proliferation, migration, and tube formation ability.^[^ ^18^ ^]^ To investigate the role of PRRC2B in endothelial cells, we examined the effect of PRRC2B knockdown on HUVEC proliferation. A hemocytometer was used to count cells at 0, 24, and 48 h, revealing that hypoxia exposure and PRRC2B knockdown inhibited cell proliferation (**Figure** **4**A). In contrast, the knockdown of PRRC2B accelerated hypoxia‐induced proliferation inhibition. To confirm this finding, we performed cell cycle analysis in HUVECs using propidium iodide staining. Flow cytometry analysis revealed that both hypoxia and PRRC2B knockdown reduced the cell number in the S/G2/M phase and decreased the HUVEC proliferation index ((S+G2/M)/(S+G2/M+G0/G1)) (Figure 4B). These results suggest that PRRC2B knockdown enhances the effect of hypoxia on endothelial cell proliferation.

We also conducted a cell wound healing assay and a cell Transwell assay to determine whether PRRC2B knockdown affects endothelial cell migration ability. The results indicated that PRRC2B knockdown promoted scratch closure compared with the control group under both normoxic and hypoxic conditions (Figure 4C). Chamber migration assay results revealed that the number of migrating HUVECs was markedly increased in the PRRC2B‐knockdown group, especially under hypoxia (Figure 4D). These results reveal that PRRC2B accelerates hypoxia‐induced cell migration. Tube formation is another intrinsic property of endothelial cells.

We assessed the effect of PRRC2B knockdown on HUVEC function using a tube formation assay. The control group and PRRC2B‐knockdown group HUVECs were seeded on a Matrigel substratum in a 48‐well plate. After 12 h of incubation in a cell culture incubator under normoxia and hypoxia, a 2–3‐fold increase in the mean number of tube formations was observed, and PRRC2B knockdown significantly enhanced hypoxia‐induced tube formation (Figure 4E). These results suggest that PRRC2B knockdown promotes hypoxia‐induced endothelial cell migration and tube formation.

### 2.3 PRRC2B Conditional Knockout Aggregates Hypoxia‐Induced Cerebral Vascular Remodeling

Cerebral oxygen supplementation is the primary factor in hypoxia‐induced brain injury. To analyze vascular remodeling in hypoxia, we developed a new 3D visualization system of mouse cerebral vasculature using the brain clarity method and light sheet microscopy (Figure S4A,B, Video S1 and S2, Supporting Information). The 3D EC lineage‐tracing (VE‐Cadherin^creER^; TD‐tomato) analysis revealed that the vessel diameter increased, but the total length was unchanged in the hypoxia‐exposed mouse hippocampus (Figure S4C–E, Supporting Information). This result indicates that hypoxia can modify the cerebral vasculature system.

**Figure 5:**
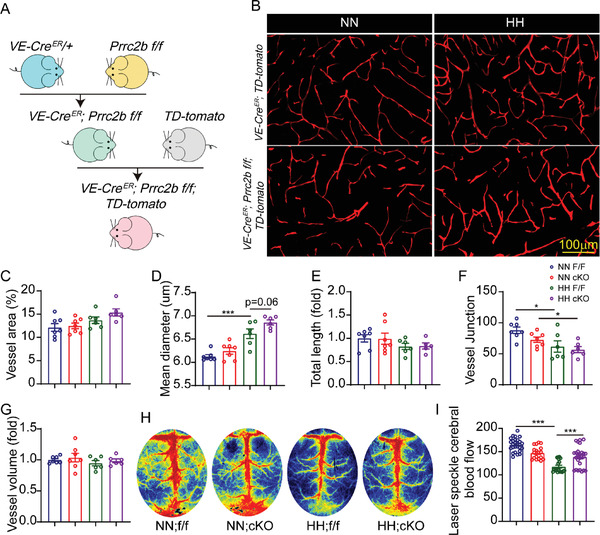
*Prrc2b* cKO modified the vasculature system and redistributed blood flow. A) Schematic illustration of the strategy for generating conditional deletion of PRRC2B in endothelial cells. B) Hippocampus vessels of control group mice (VE‐cadherin cre^ER^; TD‐tomato) and cKO mice (VE‐cadherin cre^ER^; PRRC2B flox/flox; TD‐tomato) with/without hypoxia treatment was captured by confocal microscopy. C) Statistical data of vessel area percentage, D) vessel mean diameter, E) total length of vessels, F) vessel junction numbers, and G) vessel volume in each group of mice hippocampus; (2–3 slices/mouse, *N* = 3). H) Representative image of blood flow measured by a laser speckle flowmeter. I) Statistical cerebral blood flow data were immediately measured after mouse removal from the decompression chamber and anesthesia. Each mouse recorded 4–5 data, *N* = 3 in each group (one‐way ANOVA followed by Tukey's test, *p < 0.05, **p < 0.01, ***p < 0.001).

Endothelial cell proliferation and migration are involved in vascular system remodeling. Thus, we hypothesized that PRRC2B deficiency in endothelial cells might alter the cerebral vasculature system structure under hypoxia. We crossed VE‐CadherincreER mice with Prrc2b flox mice to generate VE‐CadherincreER; Prrc2b flox/flox (shorthand notation: cKO) animals to determine the function of PRRC2B alteration in endothelial cells upon hypoxic stimulation in the brain (**Figure** 5A). Cre recombinase was activated after tamoxifen treatment, and PRRC2B was successfully depleted in cerebral endothelial cells (Figure S5A, Supporting Information). We crossed VE‐CadherincreER; Prrc2b flox/flox mice with TD‐Tomato reporter mice to generate VE‐CadherincreER; Prrc2b flox/flox; TD‐tomato and VE‐CadherincreER; TD‐tomato mice to better visualize the vasculature system. VE‐CadherincreER; Prrc2b flox/flox; TD‐tomato mice revealed no significant changes in vascular morphology compared with VE‐CadherincreER; TD‐tomato mice in normoxic conditions but showed aggravated hypoxia‐induced vessel thickening under hypoxic conditions (Figure **5**B–G and Figure S5B–G, Supporting Information). IB4 staining also revealed that PRRC2B cKO promotes hypoxia‐induced vascular remodeling (Figure S5H–J, Supporting Information). Vascular system structure changes regulate blood flow; thus, we detected blood flow in PRRC2B cKO mice. Laser speckle flow imaging revealed that hypoxia exposure decreased cerebral blood flow. In contrast, PRRC2B cKO alleviated the cerebral blood flow decrease, ensuring oxygen supply to the brain under a hypoxic environment (Figure 5H,I). These results indicate that PRRC2B cKO aggravates hypoxia‐induced vascular remodeling and blood flow distribution.

### 2.4 PRRC2B cKO Alleviates Hypoxia‐Induced Cerebral Injury

**Figure 6:**
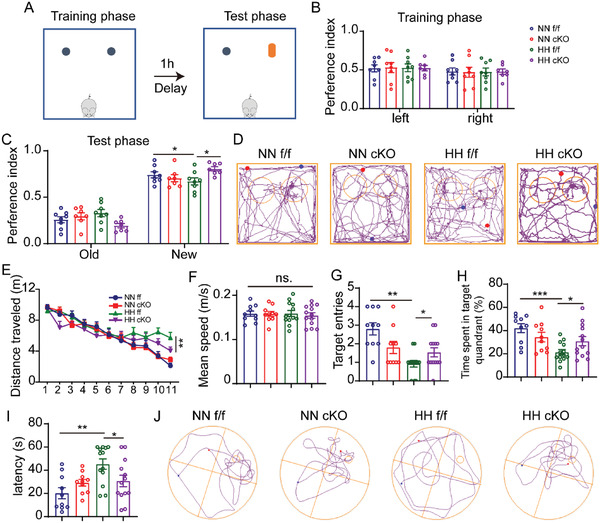
Conditional *Prrc2b* knockout in endothelial cells promotes mouse adaptation to hypoxia. A) Schematic illustration of the strategy for a novel objective recognition test. B,C) Preference indices of mice during the B) training and C) test phases of the novel object recognition test (*n* = 8, 8, 8, 7). D) Representative track plot image of mice in the novel object recognition test phase. E) Escape distance to the platform during the Morris water maze test training phase (*n* = 10, 10, 13, 13). F) Mean swimming speed of mice in the training phase. G) Number of target entries, H) time spent in the target quadrant, and I) primary escape latency in the test phase of the Morris water maze. J) Representative picture of a mouse track plot in the Morris water maze test phase (one‐way ANOVA followed by Tukey's test, *p < 0.05, **p < 0.01, ***p < 0.001).

The cKO and control mice were taken into a decompression chamber exposed to hypoxia and a hypobaric environment to determine the function of PRRC2B in mice during hypoxia. After 7 days of exposure, we performed a novel objective recognition test. The results revealed that PRRC2B cKO alleviates hypoxia‐induced cognition defects indicated by a higher preference for the new object (**Figure** **6**A–D). In the learning and memory test, after 2 days of treatment in a decompression chamber, the water maze training phase started on day 3. After each day's training phase, the mice were taken back to the hypoxia chamber. After 11 days of training, the test phase was performed without an escape platform. The results revealed that hypoxia stimulation impaired the learning ability of the mice, reflected by traveling a long distance to the escape platform without motor ability defects (Figure 6E,F). The memory ability was tested 24 h after the last training. In the test phase hypoxia exposure, the mice revealed a memory retrieval defect reflected by fewer target entries, decreased time spent in the target quadrant, long escape latency to the target, and poor escape strategies (Figure 6G–J). PRRC2B cKO mice exhibited faster escape latency, higher target entries, longer searching time in the target quadrant, and better searching strategies compared with the f/f mice in a hypoxic environment. Neuronal degeneration induced by hypoxia has been reported in hypoxia‐induced cognitive defects.^[^ ^19^ ^]^ We performed fluoro‐jade C (FJC) staining in mice brain sections to detect neuronal pathology changes. FJC staining revealed that exposure to hypoxia significantly increased the number of FJC‐positive cells, and PRRC2B cKO alleviated hypoxia‐induced neurodegeneration (Figure S6A, Supporting Information). The pathological analysis also revealed that PRRC2B cKO mice did not have splenomegaly under hypoxic conditions (Figure S6B, Supporting Information). However, no effect on blood parameters was observed (Figure S6C, Supporting Information). These results indicate that PRRC2B cKO alleviates hypoxia‐induced cerebral injury and promotes mice adaptation to hypoxic stimuli.

## 3 Discussion

Hypobaric hypoxia‐induced cognitive damage has been reported for several years; however, the underlying mechanism remains unclear.^[^ ^20^ ^]^ The cerebral vasculature system supplies oxygen to the brain, and its modulation plays an important role in hypoxia‐induced brain injury. Thus, identifying molecules and pathways involved in vascular remodeling is crucial for developing a treatment strategy.

In this study, we identified PRRC2B as a novel m6A reader. We demonstrated that PRRC2B knockdown promotes endothelial cell migration by regulating extracellular‐related gene expression, and PRRC2B regulates target gene mRNA fate in both the mRNA alternative splicing and mRNA decay process. Furthermore, we discovered that PRRC2B cKO exacerbates cerebral vascular remodeling. Thus, redistributing cerebral blood flow improves hypoxia‐induced cognitive decline in mice. This study demonstrates that a novel m6A reader protein (PRRC2B) regulates mRNA fate decisions through different mechanisms.

Previous reports reveal that PRRC2B and PRRC2C have a high‐affinity binding to m6A in oligodendrocytes; however, PRRC2B was not identified in m6A high‐affinity proteins, possibly owing to its lower expression level in oligodendrocytes. Further, PRRC2B may have a higher affinity for m6A modification RNA. Structural biological analysis using Alphafold2 revealed that PRRC2B and PRRC2A have a similar structure, and both have a low complex disorder sequence; this also indicates that PRRC2B is a potent m6A reader. Our study proves that PRRC2B binds better to m6A‐modified RNA and participates in RNA fate decisions. The PRRC2B RIP‐seq also revealed that PRRC2B binds to many intron regions (Figure 1), suggesting that it may be involved in RNA alternative splicing. Compared with m6A‐dependent PRRC2A mRNA decay regulation, PRRC2B mainly participates in mRNA alternative splicing as an m6A reader. Thus, we performed more experiments to demonstrate that PRRC2B is indeed involved in this process.

This study identified a new m6A reader protein. Previous reports reveal that m6A readers promote exon inclusion and stabilize mRNA in an m6A‐dependent manner.^[^ ^21^ ^]^ However, our results were contrary as the intersection genes of three seq data were mostly downregulated (14/19). The target genes focused on cell migration and ECM organization (COL12A1/DYPSL3) were upregulated. This result indicated a gene‐dependent role of m6A modification in RNA fate decisions. From the RNA decay assay, PRRC2B knockdown stabilized MMP14 and ADAM19 mRNA through direct binding to gene mRNA without m6A modification, indicating that PRRC2B regulation of RNA fate decision depended on the conditions involved.

Hypoxia is an external environmental stimulus for organisms; thus, epigenetic regulation is essential in hypoxic adaptation/injury. m6A modification has been reported in hypoxia‐induced angiogenesis. Yao et al. reported that METTL3 exerts its angiogenic role by regulating Wnt signaling through the m6A modification of target genes. They observed that METTL3 enhanced the translation of LRP6 and DVL1 in a YTH m6A RNA‐binding protein 1 (YTHDF1)‐dependent manner.^[^ ^10^ ^]^ Zhao et al. consistently observed that loss of m6A demethylase ALKBH5 promotes post‐ischemic angiogenesis via post‐transcriptional stabilization of WNT5A.^[^ ^22^ ^]^ In contrast, Kumari et al. demonstrated that ALKBH5 helps maintain angiogenesis in endothelial cells following acute ischemic stress via reduced SPHK1 m6A methylation.^[^ ^23^ ^]^ Based on these studies, the effect of m6A in angiogenesis varies in a context‐dependent and target gene‐specific manner.

Hypoxia‐induced vascular remodeling has been reported in lung vessels. Increased pulmonary vascular resistance and respiratory and circulatory failure result from increased wall thickness, muscle extension into normally non‐muscular arterioles, and vascular stiffening.^[^ ^7^, ^24^ ^]^ However, we observed that cerebral vascular remodeling was induced by hypoxia, and PRRC2B cKO enhanced the remodeling process, promoting mice adaptation to hypoxia. Therefore, we demonstrated the physiological implication of PRRC2B changes in endothelial cells (Figure 2). The decrease in the acute phase promotes hypoxia‐induced vascular remodeling, ensuring blood supply and recovery to the normal level and preventing excessive vascular resistance; thus, it exerts a protective role in acute hypoxia stimulation. Hypoxia‐induced blood flow changes play an important role in cognitive decline. PRRC2B cKO promotes vascular remodeling, which modifies blood flow distribution alleviating hypoxia‐induced brain injury.

## 4 Conclusion

In summary, we identified a novel m6A reader protein and demonstrated the role of PRRC2B‐mediated m6A modification in hypoxia‐induced vascular remodeling. PRRC2B mediated endothelial cell migration through elevated extracellular organization‐related gene expression. PRRC2B directly binds to gene mRNA in an m6A‐dependent manner and alters pre‐mRNA splicing and mature‐RNA decay. The results of our study provide strong evidence of the function of PRRC2B‐mediated m6A modification in hypoxia‐induced cerebral vascular physiological changes and the potential target for treating hypoxia‐induced brain injury.

## 5 Experimental Section

### Animal Housing

All C57B6 mice used in this study were housed at 22–24 °C with a 12‐h dark‐light cycle. Mice were provided with standard rodent chow and water ad libitum. All animal experiments were approved by the Institutional Animal Care and Use Committee at the Beijing Institute of Basic Medical Sciences (Beijing, China).

Eight‐ to 12‐week‐old mice were placed in a decompression chamber (model: DYC‐DWI; Fenglei, China) and were exposed to a hypobaric and hypoxic environment (O_2_: 141.69 g m^−3^, 47.2 KPa, equal to 6000 m high altitude) for 1 week. The control groups were simultaneously placed in the same chamber as the treatment groups under normoxic conditions.

PRRC2B f/f mice (generated by Dr. Fengchao Wang, National Institute of Biological Science, Beijing) were crossed with VE‐Cadherin‐Cre^ER^ transgenic mice. The resulting PRRC2B f/+; VE‐Cadherin‐CreER +/‐ mice were crossed with PRRC2B f/f mice to obtain PRRC2B f/f; VE‐Cadherin‐CreER +/‐ study subjects and their control littermates (PRRC2B f/f mice).

### Morris Water Maze Test

The water maze task was performed as previously described.^[^ ^25^ ^]^ It consisted of two phases: 1) the training phase, 12 days with a hidden platform (two trials/day), and 2) the testing phase, during which the platform was removed from the maze, lasted for 1 min, and was performed to assess the retention of previously acquired information. Mice were tracked by a video camera during training and testing. Collected data were analyzed using ANY‐maze software (Panlab, Harvard Apparatus). Statistical analyses were performed using two‐way ANOVA followed by the Bonferroni test.

### Novel Object Recognition Test

The novel object recognition test was adapted from the previous report.^[^ ^26^ ^]^ The test was performed in a clear box (50 × 50 × 20 cm). Mice were accustomed to the box for 3 days. The test included two phases. Phase 1 was a 10‐min familiarization period in which the animals were presented with two identical objects. Phase 2 was a 5‐min test in which one familiar object was replaced by a novel one. ANY‐maze software was used to record and analyze data. The times spent sniffing novel and known objects were recorded.

### Brain Clarity

Brain clarity was performed based on the Binaree Tissue Clearing Protocol (HRTC‐001, Binaree, Inc, Republic of Korea). The transparent brain was imaged using MuVi spim light sheet microscopy (Luxendo, Germany). The vessel morphology data were analyzed using Imaris software.

### Immunohistochemistry

Immunohistochemistry was performed as previously described.^[^ ^27^ ^]^ The mice's brains were post‐fixed using 4% paraformaldehyde and dehydrated with gradient sucrose (15%, 20%, and 30%) in PBS. Coronal sections (40 µm thick) were sliced with Leica CM3050S and processed for immunohistochemistry. The brain slices were immersed in PBS twice and incubated in a blocking buffer (PBS containing 0.3% Triton X‐100, 10% horse serum) for 1 h at room temperature. For vascular labeling, the slices were incubated with biotin‐labeled iso‐lectin with a 1:200 dilution (Sigma) followed by fluorescence‐conjugated streptavidin (Jackson Immunoresearch, Pennsylvania, USA) for 1 h at room temperature. Stained sections were mounted onto slides and captured by a Nikon A1 confocal microscope (Nikon, USA). The blood vessel morphological analysis was performed using Angio Tool software.^[^ ^28^ ^]^

### Tissue Harvesting and Single‐Cell Dissociation

Adult Brain Dissociation Kit was used on the endothelial cells separated from hypoxia‐and‐hypobaric‐exposure mice and control‐group mice (Miltenyi Biotec, 130107677). The brain parenchyma was harvested after mice were perfused with saline. The brain parenchyma was cut into small pieces and then digested for 30 min by gentleMACS Dissociator (130093235) in a digestion buffer. After centrifugation at 600 g for 6 min, the acquired pellet was resuspended in a debris removal buffer. After centrifugation at 3000 g for 10 min, the cells were collected from the bottom of the vial. The cells were separated by magnetic beads following red blood cell lysis; endothelial cells were isolated as CD45‐CD31+ cells.

### Cell Culture

HUVECs were cultured in Dulbecco's modified Eagle medium (Gibco, Waltham, MA, USA) supplemented with 10% fetal bovine serum, 100 U mL^−1^ penicillin, and 100 mg mL^−1^ streptomycin at 37 °C in a humidified atmosphere containing 5% CO_2_.

### Cell Proliferation Assay

HUVECs were seeded in 96‐well plates at a density of 2 × 10^3^ cells per well. Cell viability was determined at 0, 12, 24, and 48 h using the CCK8 reagent after incubating the cells for 2 h in a 37 °C humidified atmosphere containing 5% CO_2_. The density of the supernatant was measured at 450 nm with an ELISA spectrophotometer.

### Cell Migration

HUVECs were seeded in a 24‐well plate in the wound closure assay and grown to 90% confluence in 24 h. A wound was generated using a 10‐µL pipette tip, and wound closure (cell migration) was recorded by taking snapshots at 0, 12, and 24 h. The migration capacity of cells was calculated based on the width of the wound.

### Transwell Assay

The HUVECs were seeded in an 8‐µm Transwell plate (3422, Costa, ME, USA) without FBS, and the bottom chamber contained normal DMEM with 10% FBS. After 12 h, the non‐invaded cells were scraped off with a cotton swab, fixed with methanol, and stained with crystal violet. The cells were photographed under a light microscope. Thereafter, the stained cells were solubilized in 10% acetic acid and absorbance was measured at 560 nm with an ELISA spectrophotometer.

### Tube Formation

We coated 48‐well plates with Matrigel (354230, Corning, MA, USA) at room temperature. HUVECs were seeded at 2 × 10^4^ cells and incubated at 37 °C. Tube formation image was captured using Nikon Eclipse Ti2. The number of tubes was counted using Image Pro Plus software (Media Cybernetics, USA).

### Quantitative Real‐Time PCR

The experiment was performed as previously described. Total RNA was extracted from mouse brain tissue using TriZol (Thermo Fisher, USA). Reverse transcription was performed using the TransScript kit (AT311‐02, TransGen Biotech, China). Quantitative PCR was performed using SYBR Green Master Mix with High ROX (Vazyme, China) and detected by the ABI QuantStudio 3 (Thermo Fisher, USA) apparatus. The 18S RNA was used as an endogenous control. Gene expression levels were expressed as 2^−DeltaCt^. Primer sequences for qPCR are listed in Table S1, Supporting Information.

### mRNA Half‐Life Measurement

Actinomycin D (Sigma‐Aldrich) at 5 µg mL^−1^ was added to HUVECs. After 0, 0.5, 1, 2, 4, and 6 h of incubation, cells were collected, and total RNAs were isolated for qPCR assay.

### RNA‐Seq and Data Analysis

RNA sequencing was performed by LC‐Bio (Hangzhou, China). Total RNA was extracted from cultured HUVECs by using TriZol. Three sample replicates were included in each group. A stranded RNA sequencing library was constructed. The library products corresponding to 200–500 bps were enriched, quantified, and sequenced on Illumina Novaseq 6000 with the PE150 model. Raw sequencing data were first filtered by Trimmomatic (version 0.36), low‐quality reads were discarded, and the reads contaminated with adaptor sequences were trimmed. The number of reads was mapped to the reference genome of *Homo sapiens* (version: v101) using STAR software (version 2.5.3a). The expressions of transcripts were quantified as reads per kilobase of exon model per million mapped reads (RPKM). Genes with *p* < 0.05 and log2FC (fold change) ≥ 1 or log2FC ≤ −1 were considered differentially expressed. Gene Ontology (GO) analysis and Kyoto Encyclopedia of Genes and Genomes (KEGG) enrichment analysis for annotated genes were performed using KOBAS software (version: 2.1.1) with a corrected *p*‐value cut‐off of 0.05 to judge statistically significant enrichment.

### RIP‐Seq of PRRC2B in HUVECs and Data Analysis

RIP‐seq was performed on HUVEC, stably expressing 3 × FLAG‐PRRC2B‐HA by LC‐Bio (Hangzhou, China). HUVECs were collected and lysed with cell lysis buffer (150 mM NaCl, 10 mM HEPES (pH 7.6), 2 mM EDTA, 0.5% NP‐40, 0.5 mM DTT, 1% protease inhibitor cocktail, 0.4 U mL^−1^ RNasin). The 10% lysis sample was stored and named “input,” and 90% was used in immunoprecipitation reactions with anti‐HA antibodies. The RNA of the input and IP was extracted using a TRIzol reagent. RIP products were reversed transcription into a cDNA sequencing library using Invitrogen SuperScript II Reverse Transcriptase kit (CA, USA, 1896649). RNA‐seq libraries were generated with the NEBNext Ultra RNA library Prep Kit (NEB E7770S). The libraries were subjected to quality validation using the Agilent Bioanalyzer 2100 and sequenced using Illumina platforms via a 2 × 150 bp paired‐end sequencing protocol.

RNA immunoprecipitation (RIP) sequencing library preparation and data analysis were conducted by LC‐Bio (Hangzhou, Zhejiang 310018, China). Raw sequencing data were cleaned to filter adapters and low‐quality reads using FASTQC (v0.11.5), and the unique mapped reads were harvested by mapping the cleaned reads to genome *H. sapiens* (version: v101) by Bowtie 2. RIPseeker with default cut‐offs (eFDR ≤ 0.1 OR *q*‐value ≤ 0.1) was used to call peak,^[^ ^29^ ^]^ giving a robust and high‐resolution peak prediction. Homer (v4.10) was used to search motifs and analyze transcription factors.

### Photoactivatable Ribonucleotide Crosslinking and Immunoprecipitation (PAR‐CLIP)‐qPCR

HUVECs stably expressing 3 × FLAG‐PRRC2B‐HA were cultured in a medium supplemented with 4‐SU (200 µM, Sigma) for 16 h and irradiated with 0.4 J cm^−2^ of 365‐nm UV light in a cross‐linker (Analytik Jena, USA). Immuno‐precipitated by HA antibody same with RIP protocol, the protein‐RNA complexes were subjected to total RNA extraction and qPCR assay.

### m6A‐Seq and Data Analysis

m6A immunoprecipitation and library construction were modified from the published procedure.^[^ ^30^ ^]^ Fragmented and ethanol‐precipitated mRNA (6 µg) from HUVECs was incubated with 12 µg of anti‐m6A polyclonal antibody (Synaptic Systems, 202003) in an IPP buffer (150 mM NaCl, 0.1% NP‐ 40, and 10 mM Tris‐HCl [pH 7.4]) for 2 h at 4 °C. The mixture was then immunoprecipitated by incubation with 80 µL protein A beads (Sigma, P9424) at 4 °C for an additional 2 h. After washing three times, bound RNA was eluted from the beads with 0.5 mg ml^−1^ N6‐methyladenosine (Abmole, M5151) in the IPP buffer and then extracted by TRIzol. The IP RNA was reverse‐transcribed to cDNA by SuperScript II Reverse Transcriptase (Invitrogen, cat. 1 896 649, USA), which was next used to synthesize U‐labeled second‐stranded DNAs with *Escherichia coli* DNA polymerase I (NEB, cat.m0209, USA), RNase H (NEB, cat.m0297, USA) and dUTP Solution (Thermo Fisher, cat. R0133, USA). An A‐base was added to the blunt ends of each strand, preparing for ligation to the indexed adapters. Dual‐index adapters were ligated to the fragments, and size selection was performed with AMPureXP beads. After the heat‐labile UDG enzyme (NEB, cat.m0280, USA) treatment of the U‐labeled second‐stranded DNAs, the ligated products were amplified with PCR. At last, paired‐end sequencing (PE150) was performed on an Illumina Novaseq 6000 platform (LC‐Bio Technology CO., Ltd., Hangzhou, China) following the vendor's recommended protocol.

Fastp software was used to remove the reads that contained adaptor contamination, low‐quality bases, and undetermined bases with default parameters. The sequence quality of IP and input samples were verified using FastQC and RseQC. Then HISAT2 was used to map reads to the reference genome *H. sapiens* (version: v101). Peak calling and diff peak analysis were performed by R package exomePeak2, and peaks were annotated by intersection with gene architecture using R package ANNOVAR. MEME and HOMER were used for de novo and known motif finding followed by localization of the motif with respect to peak summit. StringTie was used to perform expression levels for all transcripts and genes from input libraries by calculating FPKM (total exon fragments /mapped reads (millions) × exon length (kB)). The differentially expressed transcripts and genes were selected with log2 (fold change) ≥ 1 or log2 (fold change) ≤ −1 and *p*‐value < 0.05 by R package edgeR.

### Statistical Analysis

Statistical analyses were performed using GraphPad Prism 8 software (GraphPad Prism Software). Some statistical data were normalized to the control group as indicated in the Figure legends. Data are presented as the mean ± SEM of the results of at least three replicate experiments. Two‐sided Student's *t*‐test were used to determine significant differences between groups. One‐way analysis of variance (ANOVA) was used for comparisons among multiple groups. *p* < 0.05, ** *p* < 0.01, and *** *p* < 0.001 denote the significance thresholds.

### Ethics Approval Statement

All animal experiments were approved by the Institutional Animal Care and Use Committee at the Beijing Institute of Basic Medical Sciences (Beijing, China).

## Conflict of Interest

The authors declare no conflict of interest.

## Supporting information

Supporting Information

Supplemental Video 1

Supplemental Video 2

## Data Availability

The data that support the findings of this study are available in the Supporting Information. The original seq data reported in this paper have been deposited in the National Center for Biotechnology Information sequence read archive database with links to BioProject accession ID PRJNA932863.
