# Supplementary material for: The Role of PRRC2B in Cerebral Vascular Remodeling Under Acute Hypoxia in Mice
Source: Adv Sci (Weinh). 2023 Jul 3;10(25):2300892. doi: 10.1002/advs.202300892 (PMC10477837; doi:10.1002/advs.202300892)
Supplement: Supplementary file 1 — Supporting Information [file ADVS-10-2300892-s003.pdf]

ADVANCED SCIENCE
Open Access

Supporting Information

for *Adv. Sci.*, DOI 10.1002/advs.202300892

The Role of PRRC2B in Cerebral Vascular Remodeling Under Acute Hypoxia in Mice

Shuoshuo Li, Wenyu Hu, Shenghui Gong, Ping Zhang, Jinbo Cheng, Shukun Wang, Yingyi Wang, Wenjun Shi, Qianqian Li, Fengchao Wang and Zengqiang Yuan**

Supporting Information

An m6A reader (PRRC2B) regulates cerebral vascular remodeling under acute hypoxia in mice

Shuoshuo Li, Wenyu Hu, Shenghui Gong, Ping Zhang, Jinbo Cheng, Shukun Wang, Yingyi Wang, Wenjun Shi, Qianqian Li, Fengchao Wang, Zengqiang Yuan**

Figure S1

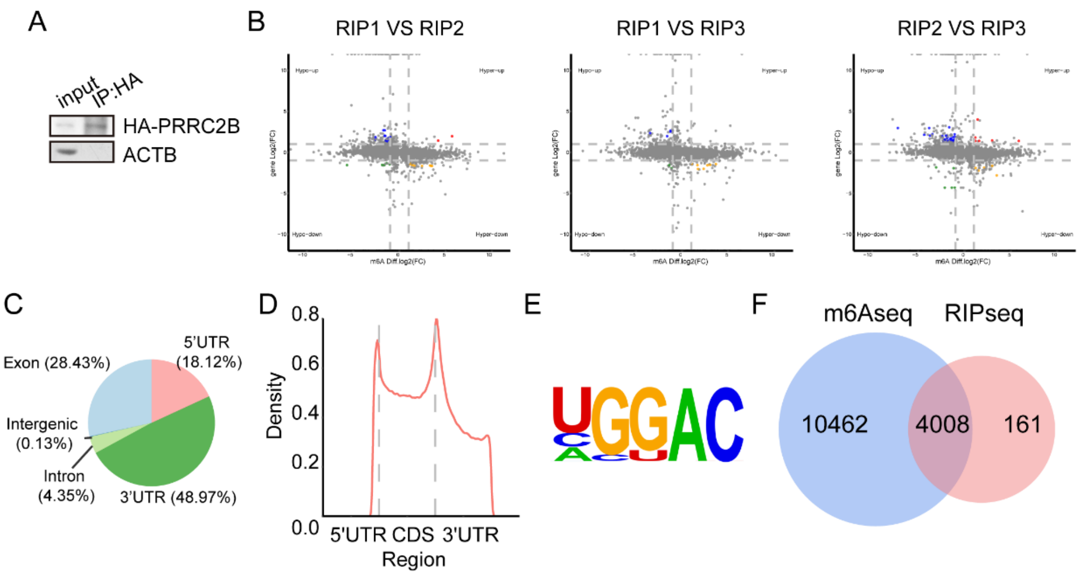

Figure S1. Related to Figure 1. PRRC2B is a novel m6A reader. A. The immunoprecipitation efficiency of PRR2CB-HA by HA antibody. B. The correlation of 3 independent repeats of m6A-seq. C. Pie chart depicting the distribution of m6A modification peaks. D. Distribution of m6A modification peaks across the length of mRNA. E. Classic m6A modification motif identified by HOMER, *p*=1e-6. F. PRRC2B binding gene containing m6A modification.

Figure S2

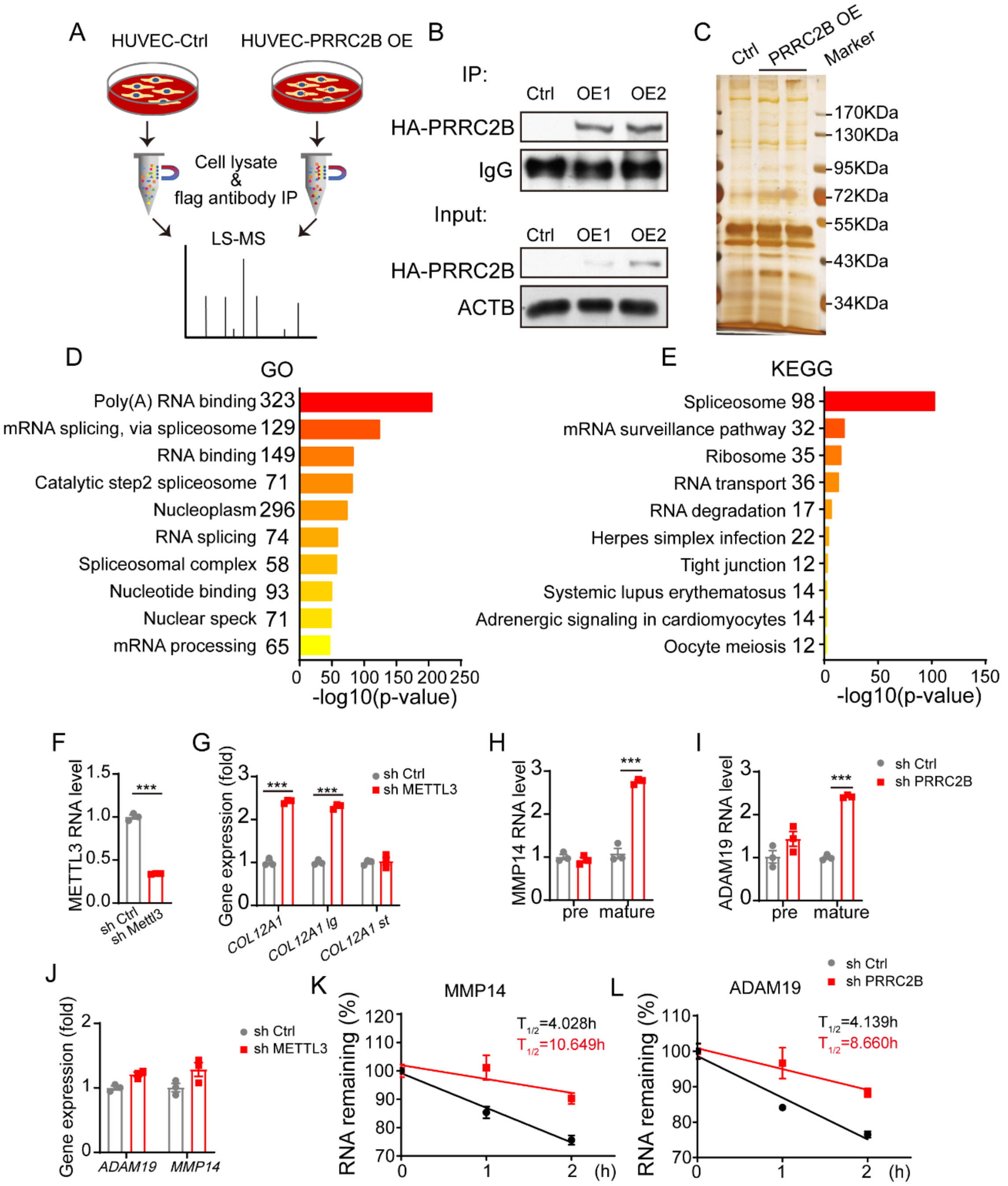

Figure S2. Related to Figure 3. PRRC2B regulates target gene fate decision. A. Schematic illustration of PRRC2B binding protein screening. B. The IP efficiency was detected by western blot. C. Sliver staining of the total proteins after immunoprecipitation. D-E. GO analysis (D) and KEEG analysis (E) of PRRC2B binding proteins enrichment pathway. F. The knockdown efficiency of si-METTL3 in HUVECs. G. The different transcriptional variants expression level in METTL3 knockdown HUVECs. H-I. The pre-mRNA and mature mRNA of MMP14 (F) and

ADAM19 (G) expression levels were detected by qPCR in the PRRC2B knockdown group. J. The mature mRNA of MMP14 and ADAM19 in METTL3 knockdown HUVECs. K-L. The mRNA decay assay of MMP14 (K) and ADAM19 (L) after actD was added to the medium. ((N=3 in QPCR experiment, Student's t-test, ***p < 0.001).

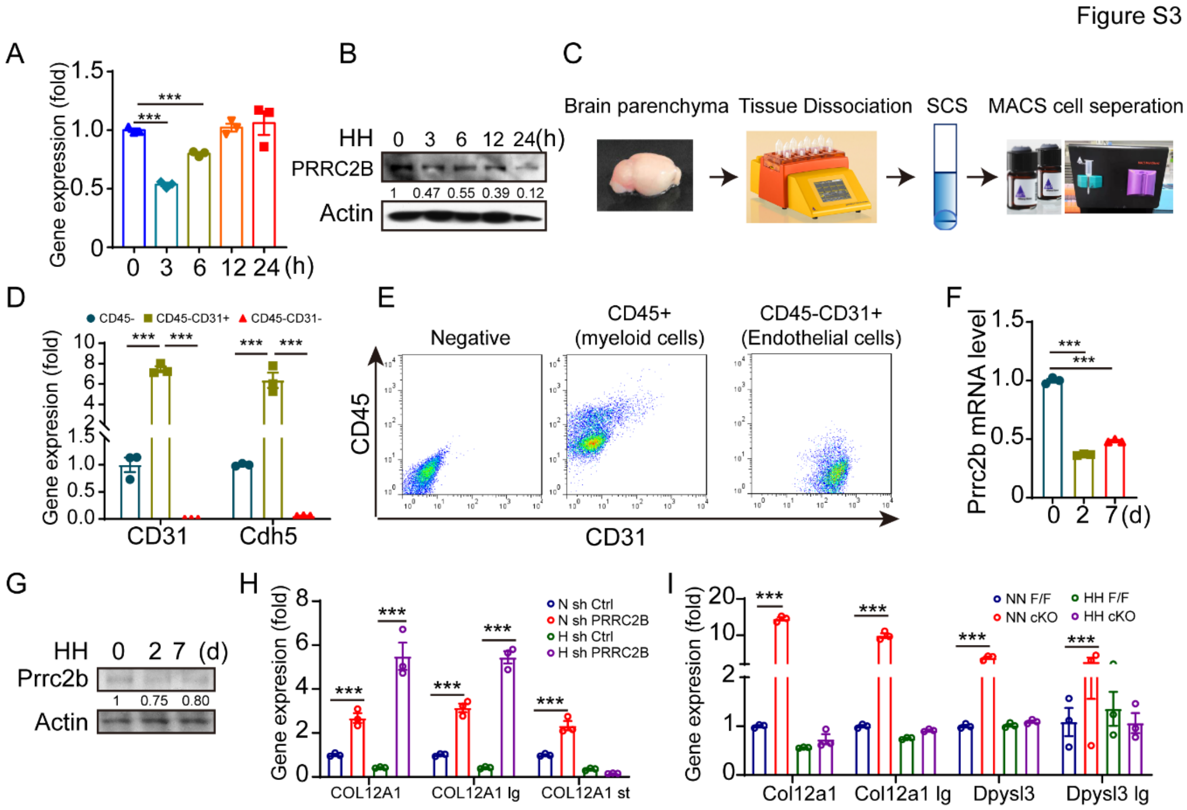

Figure S3. Related to Figure 4. PRRC2B downregulated in acute hypoxia exposure. A. The mRNA levels of PRRC2B in HUVEC across hypoxic times were detected by real-time qPCR. (Student's t-test, ***p < 0.001). B. Western blot detected the protein levels of PRRC2B in HUVEC across hypoxic times. C. Schematic of adult cerebral endothelial cells separation procedure. D. The expression level of marker genes of endothelial cells in separated cell categories. E. Flow cytometry assay of sorted cells. F-G. The mRNA level and (F) the protein level (G) of PRRC2B in mice cerebral endothelial cells upon hypoxia stimulation. H. COL12A1 transcriptional variant in hypoxia in HUVECs. I. The gene expression level in cerebral endothelial cells in Ctrl and cKO mice in hypoxia and normoxia. One-way ANOVA followed the Tukey test, ***p < 0.001, N=3.

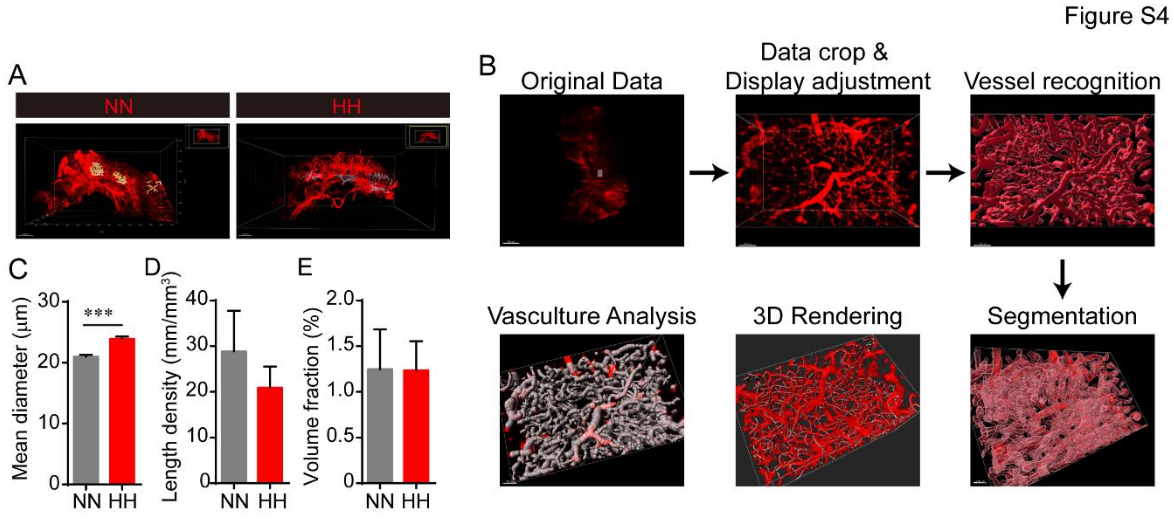

Figure S4. Hypoxia-induced cerebral vascular remodeling. A. Vascular system in the hippocampus was imaged by brain clarity methods, and blood vessels were labeled by TD-tomato fluorescence. B. The Imaris workflow of vascular data analysis captured by light sheet microscopy. C. Statistic data of blood vessel diameter in each group. D. The total vessel length per volume. E. The volume fraction percentage in normoxia and hypoxia treatment. (Student's t-test, ***$p < 0.001$).

Figure S5

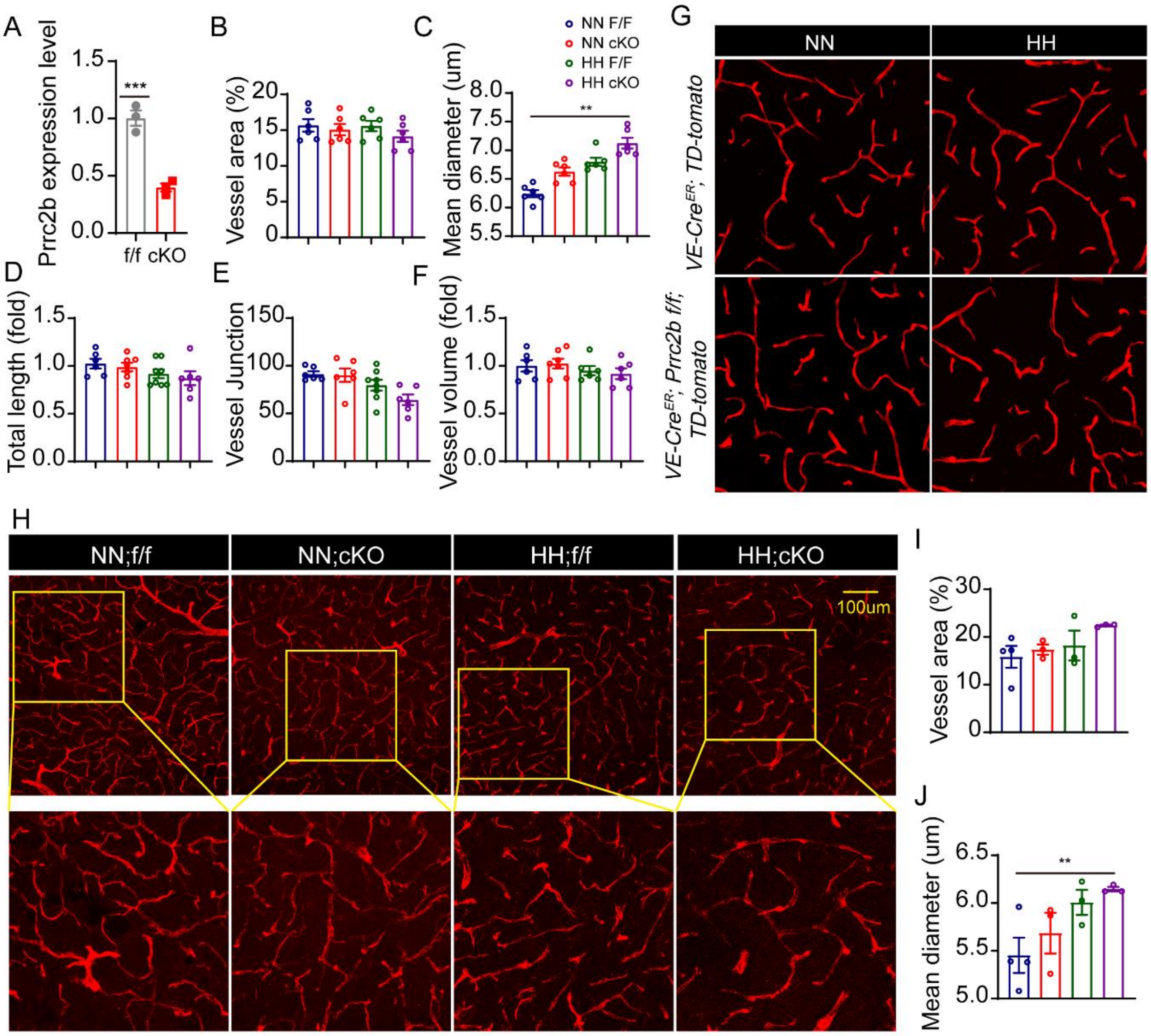

Figure S5. Related to Figure 5. Prrc2b conditional knockout aggregates hypoxia-induced vascular remodeling. A. The knockdown efficiency of *Prrc2b* in endothelial cells was determined by qPCR. B-F. Statistic data of vessel area percentage (B), vessel mean diameter (C), the total length of vessels (D), vessel junction numbers (E), and vessel volume (F) in each group of mice hippocampus. G. Representative image of cerebral blood vessels in the cortex. H. IB4 staining shows vasculature changes in the cortex, which is consistent with the results of TD-tomato-labeled mice. I-J. Statistic data of vessel area percentage (I) and vessel mean diameter (J) in IB4 staining image. (one-way ANOVA followed Tukey test, *$p < 0.05$, ***$p < 0.001$).

Figure S6

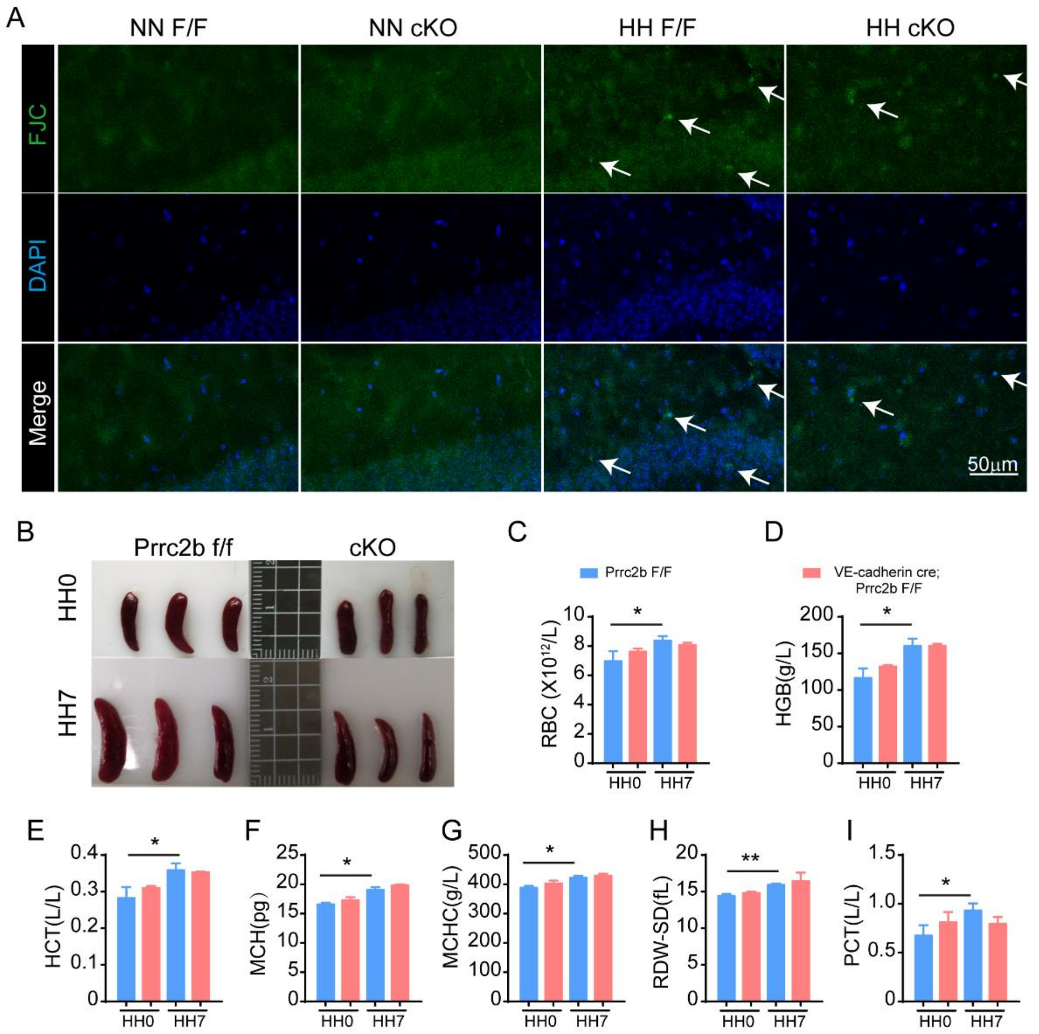

Figure S6. Related to figure 6. PRRC2B conditional knockout in endothelial cells alleviates hypoxia-induced injury. A. Representative image of FJC staining shows that hypoxia induces neurodegeneration in the hippocampus. B. PRRC2B cKO mice alleviates hypoxia-induced splenomegalia. C-I. PRRC2B cKO does not affect blood parameters. RBC: red blood cell, HGB: hemoglobin, HCT: hematocrit, MCH: Mean Corpuscular Hemoglobin, MCHC: Mean Corpuscular Hemoglobin Concentration, RDW-SD: red cell distribution width, PCT: Plateletcrit. (Student's t-test, *p < 0.01, ***p < 0.001).

Supplement table1

Category	Primer Name	Primer Sequence
RNA bait	oligo A RNA	biotin-AGAAAAGACAACCAACGAGGGACUCAUCA

sequence	probe	U
	oligo m6A RNA probe	biotin-AGAAAAGACAACCAACGAGGG^{m6}ACUCAUCAU
Knockdown sequence	si METTL3	GCCAAGGAACAATCCATTGTT
	sh PRRC2B	CCGGGCCGACAGTAAACAGAATGTTCTCGAGAACATTCTGTTTACTGTCGGCTTTTTG
QPCR primer	QhADAM19-Pre mRNA F	ACCCTCAAACCACCACACG
	QhADAM19-Pre mRNA R	GCTCACCGTAATCAGTCCTCTA
	QhMMP14-Pre mRNA F	AGTCAGCAGGTATTTATG
	QhMMP14-Pre mRNA R	TTAAGTTCTTTGGGTCA
	QhCOL12A1 trans varit st F2	TGTAAGCTCGGAGGCC
	QhCOL12A1 trans varit st R2	CACTCCATCCCTTCTG
	QhCOL12A1 trans varit lg F1	CAGGGAAAGTCGTCAA
	QhCOL12A1 trans varit lg R1	GGGCACCTTAGCAACC
	QhCOL12A1 pre-mRNA F	GAAACTCCGTCTCAAA
	QhCOL12A1 pre-mRNA R	ACCCGTGTCAACATAA
	QhDPYSL3 F	GACCGTCTCCTTATCAAGGGA
	QhDPYSL3 R	GCATCTGGAAGTGAGTATGGAC
	QhDPYSL3 trans varit1 F	CAGGAATCGGAGCAGC
	QhDPYSL3 trans varit1 R	TCGTCTTCGTGGGAGC
	QhDPYSL3 trans varit2 F	GGAGCAGAAGAAGGAG
	QhDPYSL3 trans varit2 R	AAGGAGACGGTCACTCG
	QhADAMTS1 F	TTCCACGGCAGTGGTCTAAAG
	QhADAMTS1 R	CCACCAGGCTAACTGAATTACG
	QhCOL12A1 F	CAAAGGAGGCAATACTCTCACAG
	QhCOL12A1 R	GAAGGTGCTTCAACATCGTCT
	QhADAM19 F	ACCCTCAAACCACCACACG
	QhADAM19 R	GCTCACCGTAATCAGTCCTCTA
	QhCSF2 F	TCCTGAACCTGAGTAGAGACAC

	QhCSF2 R	TGCTGCTTGTAGTGGCTGG
	QhDKK3 F	AGGACACGCAGCACAAATTG
	QhDKK3 R	CCAGTCTGGTTGTTGGTTATCTT
	QhF3 F	CCCAAACCCGTCAATCAAGTC
	QhF3 R	CCAAGTACGTCTGCTTCACAT
	QhLAMC2 F	CAAAGGTTCTCTTAGTGCTCGAT
	QhLAMC2 R	CACTTGGAGTCTAGCAGTCTCT
	QhMMP14 F	CGAGGTGCCCTATGCCTAC
	QhMMP14 R	CTCGGCAGAGTCAAAGTGG
	QhSERPINE F	GCACCACAGACGCGATCTT
	QhSERPINE R	ACCTCTGAAAAGTCCACTTGC
	QhMETTL3 F	TTGTCTCCAACCTTCCGTAGT
	QhMETTL3 R	CCAGATCAGAGAGGTGGTGTAG
	QmCDH5 F	CACTGCTTTGGGAGCCTTC
	QmCDH5 R	GGGGCAGCGATTCATTTTTCT
	QmCD31 F	CTGCCAGTCCGAAAATGGAAC
	QmCD31 R	CTTCATCCACCGGGGCTATC
	QmPrrc2b 1F	GGCCGGTCCAAACTCTGTC
	QmPrrc2b 1R	CCACTCCTCGTTGGCATGA
	QmCol12a1 F	AAGTTGACCCACCTTCCGAC
	QmCol12a1 R	GGTCCACTGTTATTCTGTAACCC
	QmCol12a1 lg F	CGCCAGTGCTGCTTAC
	QmCol12a1 lg R	TCTCCACCCTCCTTTG
	QmCol12a1 st F	GTGCCAGCATCCCATAC
	QmCol12a1 st R	CGCCGTAGTCCTCGTATT
	QmDpysl v2-st F	TAGCAGCGAGGCAGAC
	QmDpysl v2-st R	TTCTCCCTCCCTTGATTAGAAGACG
	QmDpysl v3-lg F	AGCCGCTGTCGCTTGA
	QmDpysl v3-lg R	TGCCGTGGGACCTGAT
	QmDpysl F	CCGTCTTCTAATCAAGGGAGGG
	QmDpysl R	AATCGTCCACTGTGGTCATTC
